# Schwann cells modulate nociception in neurofibromatosis 1

**DOI:** 10.1172/jci.insight.171275

**Published:** 2024-01-23

**Authors:** Namrata G.R. Raut, Laura A. Maile, Leila M. Oswalt, Irati Mitxelena, Aaditya Adlakha, Kourtney L. Sprague, Ashley R. Rupert, Lane Bokros, Megan C. Hofmann, Jennifer Patritti-Cram, Tilat A. Rizvi, Luis F. Queme, Kwangmin Choi, Nancy Ratner, Michael P. Jankowski

**Affiliations:** 1 Department of Anesthesia, Division of Pain Management, Cincinnati Children’s Hospital Medical Center, Cincinnati, Ohio, USA.; 2 Graduate Program in Neuroscience, University of Cincinnati College of Medicine, Cincinnati, Ohio, USA.; 3 Division of Cancer Biology and Experimental Hematology and; 4 Pediatric Pain Research Center, Cincinnati Children’s Hospital Medical Center, Cincinnati, Ohio, USA.; 5 Department of Pediatrics, University of Cincinnati College of Medicine, Cincinnati, Ohio, USA.

**Keywords:** Neuroscience, Calcium, Mouse models, Pain

## Abstract

Pain of unknown etiology is frequent in individuals with the tumor predisposition syndrome neurofibromatosis 1 (NF1), even when tumors are absent. Nerve Schwann cells (SCs) were recently shown to play roles in nociceptive processing, and we find that chemogenetic activation of SCs is sufficient to induce afferent and behavioral mechanical hypersensitivity in wild-type mice. In mouse models, animals showed afferent and behavioral hypersensitivity when SCs, but not neurons, lacked *Nf1*. Importantly, hypersensitivity corresponded with SC-specific upregulation of mRNA encoding glial cell line–derived neurotrophic factor (GDNF), independently of the presence of tumors. Neuropathic pain-like behaviors in the NF1 mice were inhibited by either chemogenetic silencing of SC calcium or by systemic delivery of GDNF-targeting antibodies. Together, these findings suggest that alterations in SCs directly modulate mechanical pain and suggest cell-specific treatment strategies to ameliorate pain in individuals with NF1.

## Abstract 2

<p>Schwann cells underlie pain development in models of neurofibromatosis 1 through calcium-modulated GDNF signaling.</p>

## Graphical abstract

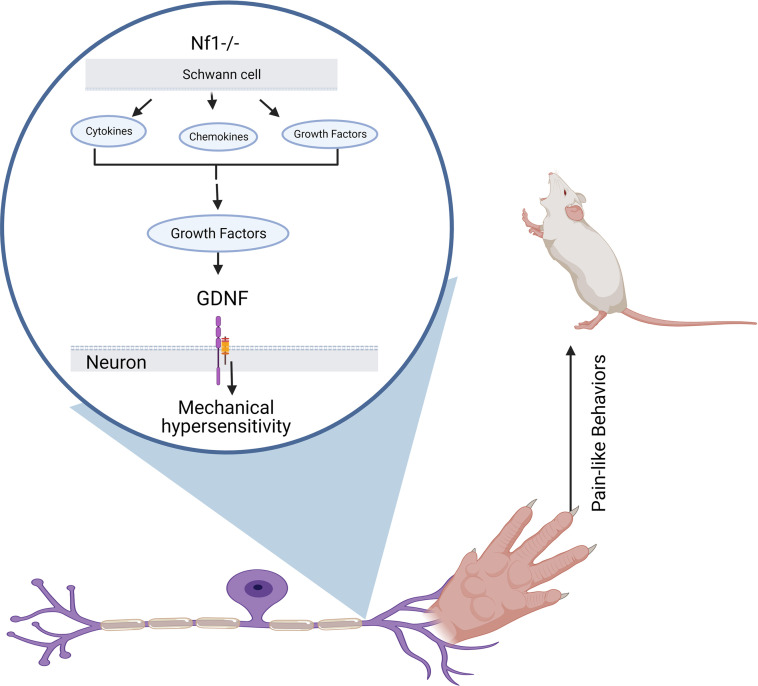

## Introduction

Primary afferent neurons transduce sensory information from the peripheral nervous system (PNS) to the central nervous system (1–4). Several pieces of recent evidence suggest a pivotal role for non-neuronal cells, including glia, in modulating the complex communication among cell types to regulate somatosensation (5–8). Schwann cells (SCs) in particular have been shown to play a crucial role in nociceptive processing in the periphery. SCs are themselves mechanically sensitive and can contribute to somatosensation (5).

SC diseases often present with pain as a chief complicating factor (9, 10). Neurofibromatosis 1 (NF1) is a genetic disorder present in approximately 1/3,000 live births (11–13). NF1 is a multisystem disorder with widespread complications, which can include multiple flat, light-brown patches of skin pigmentations (café-au-lait spots); skinfold freckling; nerve tumors (cutaneous neurofibromas) under the skin; and small nodules in the iris (Lisch nodules), as well as motor and cognitive dysfunction, bone abnormalities, and predisposition to other tumor types. At least half of individuals with NF1 develop plexiform neurofibromas in the peripheral or cranial nerves, which can transform into malignant peripheral nerve sheath tumors (12, 14–18). Plexiform neurofibromas are a debilitating complication of NF1, as they can cause disfigurement and/or functional impairment. These nerve tumors present a major challenge for therapy (19), as the only curative strategy available is surgical resection, which is often not possible because of tumor-integrated nerves (20). As a result, tumor-associated pain can be a major debilitating symptom in patients with NF1 (21). However, patient-reported pain, which is described as neuropathic in nature and can be moderate to severe in its intensity, decreasing quality of life, often precedes or can be independent of tumor formation (22).

NF1 is characterized by loss of the *Nf1* gene, which produces neurofibromin, a negative regulator of Ras/GTP signaling that modulates cell growth (23–25). There are mutations in both *Nf1* alleles in neurofibromas and neurofibroma SCs (12, 26–29). Complete *Nf1* loss of function in SCs correlates with neurofibroma formation. Therefore, SCs and/or their precursors are the known pathogenic cells in neurofibroma development (12, 26, 30). In contrast, most nonglial cells, including sensory neurons, are wild-type (in somatic mosaic patients or sporadic neurofibroma) or heterozygous for *Nf1* mutations (in most individuals with NF1) (16, 17, 31–34).

Mice that are *Nf1* haploinsufficient (Nf1^+/–^), and a few other rodent models of NF1, have been used to model NF1-related hypersensitivity and pain; however, none of these models recapitulate all of the features of NF1 (35–38). Genetically engineered mice that carry a homozygous deletion of *Nf1* in SCs and SC precursors (SCPs) causes spontaneous tumor formation over time and have become an essential tool to study NF1 tumorigenesis (39, 40). The potential contribution of SCs lacking *Nf1* to the development of pain, however, has not been studied.

Neurofibromas contain elevated levels of signaling molecules that include chemokines, cytokines, and various growth factors (14). Elevated factors are known to play a prominent role in the onset of pain in many neuropathic pain-like conditions (6, 41–44). SCs also modulate pain perception by releasing specific algesic neurotrophic factors and cytokines/chemokines (41, 45). While *Nf1*-mutant SCs express higher levels of factors than wild-type (WT) cells, the contribution of SC factor release in pain development in NF1 remains unclear. Here, we found that SCs are primary contributors to hypersensitivity in a mouse model of NF1. Pain-like behaviors are observed prior to tumor formation and are regulated by enhanced glial cell line–derived neurotrophic factor (GDNF) expressed by SCs.

## Results

### Deletion of Nf1 in SCs but not sensory neurons causes mechanical hypersensitivity.

**Figure 1:**
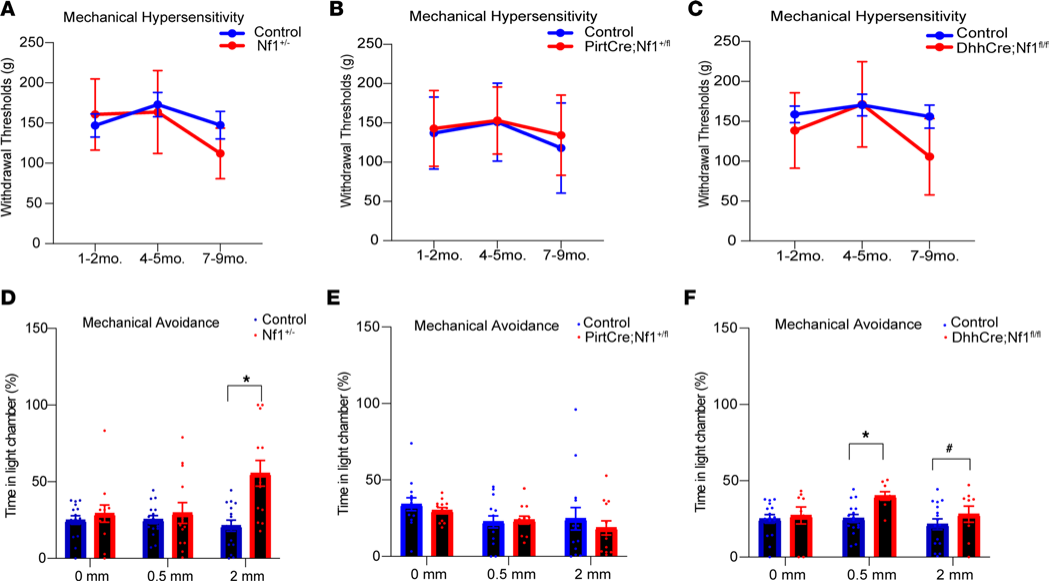
SC-specific knockout of *Nf1* leads to mechanical hypersensitivity. (**A**) *Nf1* haploinsufficient mice (Nf1^+/–^) do not show mechanical hypersensitivity using the Randall-Selitto (R-S) assay (*n* = 12 control; *n* = 13 mutant). (**B**) Deletion of *Nf1* in sensory neurons (PirtCre Nf1^+/fl^) does not cause any mechanical hypersensitivity using R-S (PirtCre Nf1^+/fl^; *n* = 17 control; *n* = 17 mutant). (**C**) Mice with SC-specific *Nf1* deletion (DhhCre Nf1^fl/fl^) show a trend toward reduced mechanical withdrawal thresholds (*n* = 20 control; *n* = 12 mutant; *P* < 0.077 vs. time-matched littermate controls; 2-way repeated measures [RM] ANOVA, Tukey’s post hoc test; mean ± SEM). (**D**) Using MCA assay, Nf1^+/–^ mice prefer to spend more time during the assay exposed to an aversive light stimulus compared with a noxious mechanical stimulus. (*P < 0.05 vs. controls, 1-way ANOVA with Tukey’s post hoc; mean ± SEM). (**E**) PirtCre Nf1^+/fl^ mice did not show any significant difference in time spent in either light or dark chambers. (**F**) DhhCre Nf1^fl/fl^ mice display increased mechanical avoidance even with smaller spikes present versus littermate controls. (*P < 0.05, ^#^*P* < 0.05 vs. controls 1-way ANOVA with Tukey’s post hoc; mean ± SEM.)

Our reports have suggested that SCs play an important role in nerve integrity and tumor development in NF1 (40). We therefore wanted to determine whether, in a disease model with altered SC biology, altered SCs also contributed to pain, a debilitating symptom in patients with NF1 (19, 46). To determine if *Nf1* deletion in neurons and/or SCs causes hypersensitivity, we evaluated behavioral responsiveness in a sensory neuron *Nf1*-mutant mouse (PirtCre Nf1^+/fl^) or in an SC-specific *Nf1* mutant (desert hedgehog–Cre Nf1^fl/fl^, DhhCre Nf1^fl/fl^). Use of these Cre lines coordinated the timing of deleting *Nf1* from neurons and SCs to about E11–E12 (14, 40). To recapitulate cell mutational status in most individuals with NF1 (40, 47), we assayed mice with heterozygous deletion in sensory neurons (PirtCre Nf1^+/fl^) and homozygous deletion in SCs (DhhCre Nf1^fl/fl^). Finally, we used mice in which neurons, SCs, and all other cell types were haploinsufficient for *Nf1* (i.e., Nf1^+/–^). Using standard evoked cutaneous mechanical hypersensitivity assays (Randall-Selitto test) on the hairy skin of the hind paw (48), similar to previous reports in uninjured mice (49), haploinsufficient knockout mice showed no significant difference in responsiveness over time when compared to littermate controls (Figure 1A). Mice with 1 copy of *Nf1* deleted in sensory neurons (PirtCre Nf1^+/fl^) also did not show mechanical hypersensitivity by Randall-Selitto testing at any time point (Figure 1B). In contrast, DhhCre Nf1^fl/fl^ mice (40) displayed a trend toward mechanical hypersensitivity at the latest time points tested in the Randall-Selitto assay (Figure 1C).

We then tested animals in a more operant task, the mechanical conflict avoidance (MCA) assay (50, 51). This test allows the animal to freely choose between bright light as an aversive stimulus or noxious mechanical stimulation of the paws. Utilizing this method allows us to measure avoidance behavior in mice upon increasing levels of noxious stimuli. In the MCA test, 4-month-old Nf1^+/–^ animals displayed enhanced mechanical avoidance, indicating that this assay is sensitive for assessing pain-like behaviors in models of NF1 (Figure 1D). However, using the MCA assay in 4-month-old PirtCre Nf1^+/fl^ mice, no differences in mechanical hypersensitivity were observed compared to controls (Figure 1E). Importantly, DhhCre Nf1^fl/fl^ mice at 4 months did show significantly increased mechanical avoidance (Figure 1F). Combining the heterozygous deletion of *Nf1* in both sensory neurons and SCs did not alter mechanical avoidance in the MCA assay (Supplemental Figure 1A; supplemental material available online with this article; https://doi.org/10.1172/jci.insight.171275DS1), suggesting that other cell types may contribute to effects in Nf1^+/–^ mice. Of note, when assessing intercrossed PirtCre Nf1^+/fl^ and Nf1^fl/fl^ mice, only 1 of 37 animals from these litters had both copies of *Nf1* deleted from sensory neurons (PirtCre Nf1^fl/fl^), suggesting issues with survivability with complete deletion of *Nf1* from primary afferents at embryonic stages.

Since previous work has shown that deletion of *Nf1* in SCs causes disruptions in nerve structure, we also assessed Remak bundle integrity (groups of unmyelinated axons wrapped by an SC at 4–5 months of age, a time when tumors are not yet present in the lumbar dorsal root ganglia [DRGs] or saphenous nerves) (40). The saphenous nerve from Nf1^+/–^ and PirtCre Nf1^+/fl^ mice displayed no significant alterations in Remak bundle structure (Supplemental Figure 1, B–E) at 4–5 months. Remak bundles contain C-fibers, axons that convey multimodal sensory information, including pain and temperature information. In the DhhCre Nf1^fl/fl^ mice, the disruption of the Remak bundles increased significantly during this time frame as previously described for this model system (14, 40). Together, this suggests that SCs play an important role in the onset of hypersensitivity in NF1 before tumor formation but during Remak bundle disruption.

### Mechanical but not thermal hyperresponsiveness is observed in primary afferents of mice with SC deletion of Nf1.

**Figure 2:**
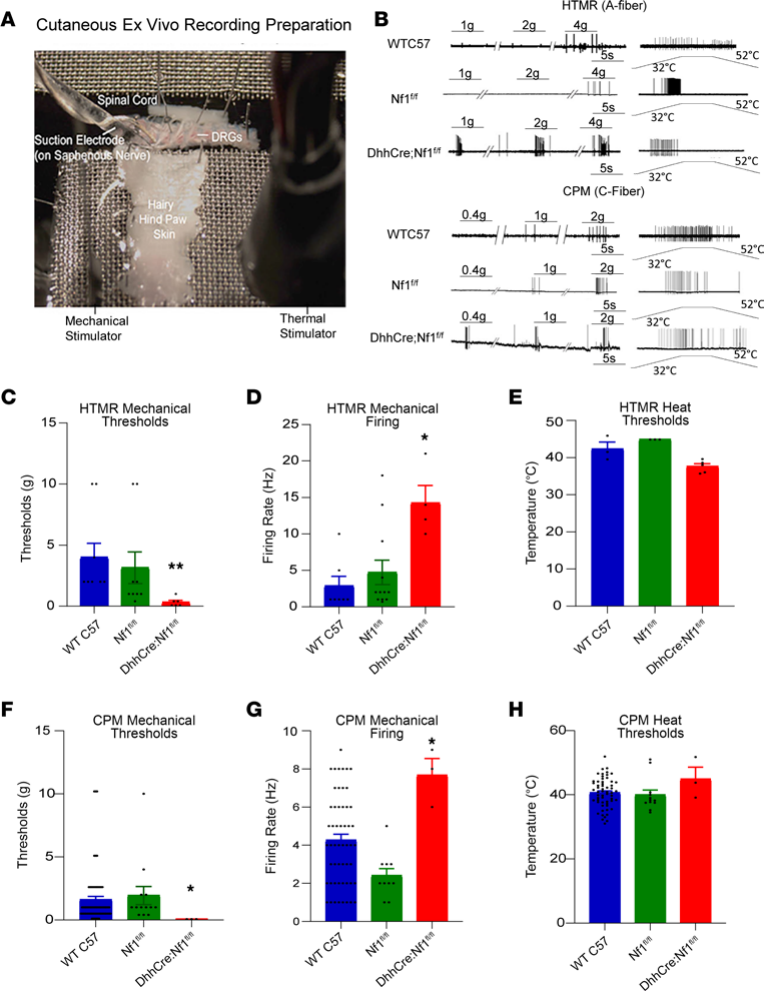
Sensitization of high-threshold mechanoreceptors and polymodal C-fibers in DhhCre Nf1^fl/fl^ mice as assessed with ex vivo recording. (**A**) Representative image of the ex vivo electrophysiological recording preparation. (**B**) Firing pattern of high-threshold mechanoreceptors (HTMRs) or A-fibers and polymodal C-fibers (CPMs) in WT C57BL/6 (C57) controls and Nf1^fl/fl^ and DhhCre Nf1^fl/fl^ mice at 4–5 months of age. (**C**) HTMRs from DhhCre Nf1^fl/fl^ mice showed a significant reduction in mechanical thresholds compared with control HTMRs (WT C57, *n* = 9; Nf1^fl/fl^, *n* = 9, mutant, *n* = 7; *P < 0.05 vs. Nf1^fl/fl^. **P < 0.01, vs. Nf1^fl/fl^, 1-way ANOVA with Tukey’s post hoc; mean ± SEM; total no. of cells, WT C57, *n* = 45; Nf1^fl/fl^, *n* = 57, mutant, *n* = 37). (**D**) Firing rates of HTMRs showed the increased firing to mechanical stimuli in DhhCre Nf1^fl/fl^ mice when compared with controls (*P < 0.05 vs. Nf1^fl/fl^ 1-way ANOVA with Tukey’s post hoc; mean ± SEM). (**E**) HTMRs showed no change in heat thresholds. (**F**) CPMs in DhhCre Nf1^fl/fl^ mice also showed reduced mechanical thresholds compared with controls (WT C57, *n* = 14; Nf1^fl/fl^, *n* = 14, mutant, *n* = 8; *P < 0.05 vs. Nf1^fl/fl^, 1-way ANOVA with Tukey’s post hoc; mean ± SEM). (**G**) DhhCre Nf1^fl/fl^ mice also showed the increased firing rate of CPMs (*P < 0.05 vs. Nf1^fl/fl^, 1-way ANOVA with Tukey’s post hoc; mean ± SEM). (**H**) CPMs in DhhCre Nf1^fl/fl^ mice showed no change in heat thresholds.

Our behavioral data suggest that SCs are key players in NF1-related hypersensitivity. Changes in neuronal firing can accompany pain onset. Therefore, we determined whether deleting *Nf1* from SCs alters primary afferent responsiveness, using an ex vivo preparation that contained hairy skin, saphenous nerve, DRG, and spinal cord (52, 53) (Figure 2, A and B). We found that in the DhhCre Nf1^fl/fl^ mice, the myelinated HTMRs displayed a significant reduction in mechanical thresholds (Figure 2, B and C) and an increase in firing to mechanical stimulation of their receptive fields compared with WT C57BL/6 and Nf1^fl/fl^ controls (Cre-negative) but showed no change in heat responsiveness (Figure 2, B, D, and E). CPMs in the DhhCre Nf1^fl/fl^ mice also displayed reduced mechanical thresholds and enhanced firing rates in response to mechanical stimuli but no change in heat sensitivity compared to controls (Figure 2, B and F–H). No significant changes in response properties were observed in other neuronal subtypes between groups (Supplemental Figure 2), including low-threshold mechanoreceptors (Supplemental Table 2). These results suggest that SCs play a role in the sensitization of adjacent sensory neurons to mechanical stimuli, which could underlie pain-like behaviors in NF1.

### Chemogenetic activation of SCs induces peripheral hypersensitivity.

**Figure 3:**
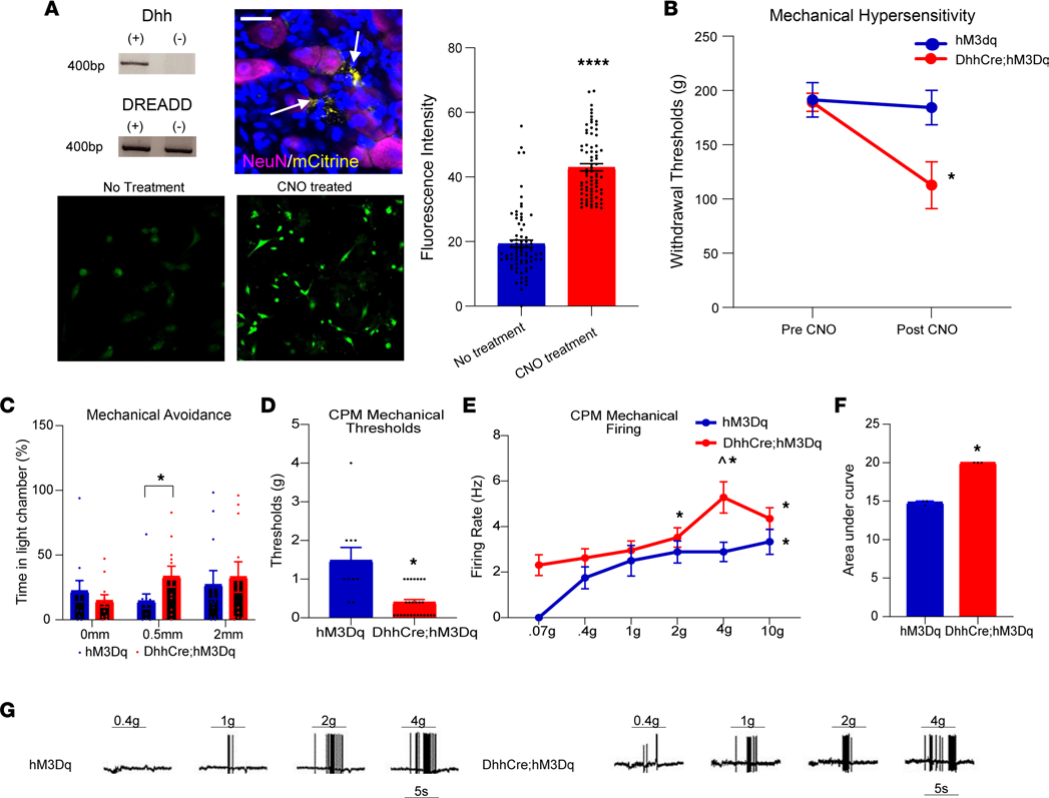
Chemogenetic activation of SCs induces peripheral hypersensitivity. (**A**) DhhCre hM3Dq mice expressing DREADD reporter (top left) and mCitrine in SCs (yellow) surrounding putative NeuN^+^ neurons (purple) in DRGs (top right). SC cultures from DhhCre hM3Dq mice treated with CNO (40 μM) display enhanced calcium fluorescence (Fluo-4) compared with untreated cultures (bottom left and right, and bar graph, scale bar = 100 μm) (****P < 0.0001 vs. no treatment, *t* = 14.44, df = 148; *t* test; mean ± SEM). (**B**) Treatment of DhhCre hM3Dq mice (*n* = 5) for 7 days with CNO (2 mg/kg, i.p. once/d) induces mechanical hypersensitivity compared with CNO-treated controls (*n* = 8) by R-S (*P < 0.05 vs. hM3Dq after CNO, 2-way ANOVA with Tukey’s post hoc; mean ± SEM). (**C**) Similar results are also seen using the MCA assay (*P < 0.05 vs. hM3Dq after CNO, 1-way ANOVA with Tukey’s post hoc; mean ± SEM). (**D**) Ex vivo recording of saphenous afferents indicates reduced mechanical thresholds in CPM fibers in CNO-treated DhhCre hM3Dq mice (*n* = 12 CPMs) compared with controls (*n* = 12 CPMs) (*P < 0.05 vs. hM3Dq, 1-way ANOVA with Tukey’s post hoc; mean ± SEM). (**E**) Enhanced firing over increasing forces (stimulus encoding) observed in control CPMs was *not* found in the DhhCre hM3Dq CPMs. ^*P* = 0.0038, DhhCre hM3Dq vs. hM3Dq. (**F**) Area under the curve for firing rates for CPMs. *P < 0.05, 1-way (**A**, **C**, **D**, and **F**) or 2-way RM (**B** and **E**) ANOVA with Tukey’s post hoc as appropriate; mean ± SEM. (**G**) Example firing patterns of CPM neurons from DhhCre hM3Dq and hM3Dq mice after 7days of CNO injections.

Recent studies showed that optogenetic stimulation of SCs can modulate nociception from the skin (5). We tested whether direct activation of SCs through GPCRs might also regulate peripheral sensitization. Because SCs use calcium as a major source of intracellular signaling, we utilized a transgenic mouse that expressed a Cre-driven Gq-coupled designer receptor exclusively activated by designer drugs (DREADD) in SCs (DhhCre hM3Dq) to allow for the artificial manipulation of SC calcium signaling (41, 54, 55). Also, utilizing a chemogenetic strategy allows us to initiate a physiologically relevant “activation” in most SCs, rather than in an isolated receptive field stimulated by light using optogenetics. Further, since SCs mutant for *Nf1* display enhanced ATP-mediated calcium responses (56), this allows us to compare results from DREADD experiments with the SC-specific NF1 mouse models using the same Cre driver. We verified that chemogenetic manipulation of primary SCs in vitro effectively increased SC calcium levels upon treatment with the designer drug clozapine-N-oxide (CNO) (57, 58). We also verified that, as expected for the Dhh-Cre driver (40), in our transgenic mice the DREADD was expressed in satellite glial cells, SCs that surround neurons in the DRGs, and nerve SCs but not DRG neurons (Figure 3A). We then performed a dose response analysis on mice treated with CNO once daily for up to 7 days to determine if SC calcium modulation might alter mechanical withdrawal thresholds in vivo (Supplemental Figure 3A). We found that elevated calcium via delivery of CNO for 7 days to 4-month-old DhhCre hM3Dq mice in vivo was sufficient to decrease mechanical withdrawal thresholds as assessed using Randall-Selitto mechanical hypersensitivity testing (Figure 3B). This treatment regimen also caused the animals to avoid a noxious mechanical stimulus in the MCA assay (Figure 3C).

We then tested if enhanced calcium in SCs affects adjacent sensory neurons in the DRG and peripheral nerve using ex vivo recording. We found that CPMs in the CNO-treated DhhCre hM3Dq mice were sensitized to mechanical (Figure 3D) but not heat stimulation (Supplemental Figure 3F) of their receptive fields (RFs) compared with CNO-treated controls. A known characteristic of nociceptors is their ability to encode stimulus intensity (59). An intriguing finding from our ex vivo recordings in DhhCre hM3Dq mice was that CPM neurons appeared to lose this encoding capacity for mechanical stimuli, in that they reached near-peak firing rates at the lowest thresholds tested (Figure 3, E–G). Significant effects of SC-mediated sensitization were specific to the CPM neuron subpopulation (Supplemental Figure 3, B–E).

Previous work has shown that SCs are sources of a variety of growth factors and cytokines that are considered algesic and that *Nf1*-mutant SCs produce increased levels of such factors (41, 60–62). We performed a small screen of factors known to be produced by Nf1^–/–^ SCs using RNA from the DRGs/nerve roots of DhhCre hM3Dq mice treated with CNO. We found that enhancing calcium in SCs caused an upregulation of mRNAs encoding several growth factors and cytokines that could affect peripheral sensitivity, including GDNF, nerve growth factor (NGF), and monocyte chemoattractant protein 1 (MCP1) (Table 1). Together, these data suggest that alterations in GPCR signaling in SCs are sufficient to alter production of SC factors known to alter specific sensory neuron populations that modulate mechanical responsiveness.

### Inhibition of enhanced SC calcium in DhhCre Nf1^fl/fl^ hM4Di mice reduces mechanical hypersensitivity.

**Figure 4:**
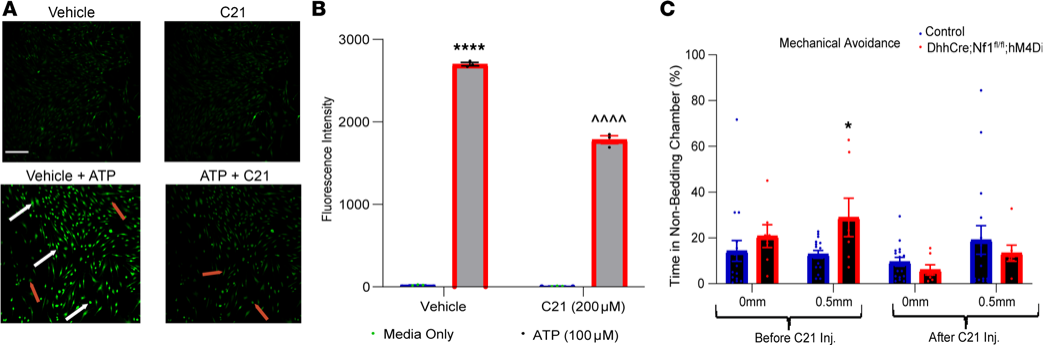
Chemogenetic inhibition of SCs suppresses mechanical hypersensitivity in DhhCre Nf1^fl/fl^ hM4Di mice. (**A**) In isolated SCs from sciatic nerves of DhhCre Nf1^fl/fl^ hM4Di mice, no significant changes in calcium are detected in SCs treated with vehicle (DMSO) (top panel, left). Calcium release is increased upon addition of ATP (100 μM with vehicle) (bottom panel, left). No significant changes in calcium are detected in SCs treated with compound 21 (C21) alone. Inhibition of ATP-induced calcium is observed, however, with C21 in SCs isolated from DhhCre Nf1^fl/fl^ hM4Di mice (bottom panel, right) (scale bar = 100 μm). White arrow indicates cells displaying green fluorescence, and the red arrows mark the absence of fluorescence within cells in the respective images (bottom, left and right). (**B**) Quantification of fluorescence intensity from SCs depicting changes in calcium release from conditions outlined in **A** (****P < 0.0001 ATP with vehicle vs. ATP with C21 only, and ^^^^*P* < 0.0001 C21 vs. ATP with C21, 2-way ANOVA with HSD post hoc; mean ± SEM). (**C**) DhhCre Nf1^fl/fl^ hM4Di mice display increased mechanical avoidance even with smaller spikes present vs. littermate controls (*n* = 16 control, *n* = 7 mutant, *P < 0.05 vs. controls 2-way ANOVA, Tukey’s post hoc; mean ± SEM), before C21 injection, but after 7 days of C21 injection (i.p.), mechanical avoidance is reduced to control levels.

Previous work has shown that Nf1^–/–^ SCs have significantly elevated calcium responses to stimulation with ATP (56). ATP acts through GPCRs on the SC surface, which couple to increases in calcium via activation of downstream signaling, through small G proteins. We tested if we could reverse mechanical hypersensitivity observed in the NF1 mouse model by use of an inhibitory DREADD in SCs (DhhCre Nf1^fl/fl^ hM4Di). This DREADD suppresses calcium intracellularly by activation of Gi (63). We verified that SCs isolated from DhhCre Nf1^fl/fl^ hM4Di mice displayed enhanced calcium responses to ATP stimulation. Treatment of SC cultures with the DREADD agonist compound 21 (C21) significantly inhibited the ATP-induced calcium response (Figure 4, A and B). C21 was used in these experiments to avoid potential nonspecific effects of high-dose CNO, which are often required for activation of the inhibitory DREADD in vivo (64). In the MCA assay, prior to C21 treatment, DhhCre Nf1^fl/fl^ hM4Di mice displayed the expected increase in mechanical avoidance. However, after treating these mice with C21 for 7 days, even without any noxious mechanical stimulus added, mice spent equal amounts of time in the light and dark chambers, indicating that inhibition of SCs may affect light sensitivity (Supplemental Figure 4). We therefore modified this assay to avoid the use of light as an aversive stimulus. Instead, we allowed mice to perform the MCA task when all chambers were dark. We provided one side with home cage bedding. Normal mice choose to spend more time in the home-bedding chamber in this assay; however, DhhCre Nf1^fl/fl^ hM4Di mice, prior to C21 treatment, spent less time crossing the noxious mechanical stimulus in order to reach the home-bedding chamber. After 7 days of C21 treatment, however, these same mice showed no differences compared to controls (Figure 4C). These results strongly suggest that enhanced calcium signaling in SC is a major driver of the pain-like behavior in this mouse model of NF1.

### SC-specific deletion of Nf1 alters gene expression in DRGs.

**Figure 5:**
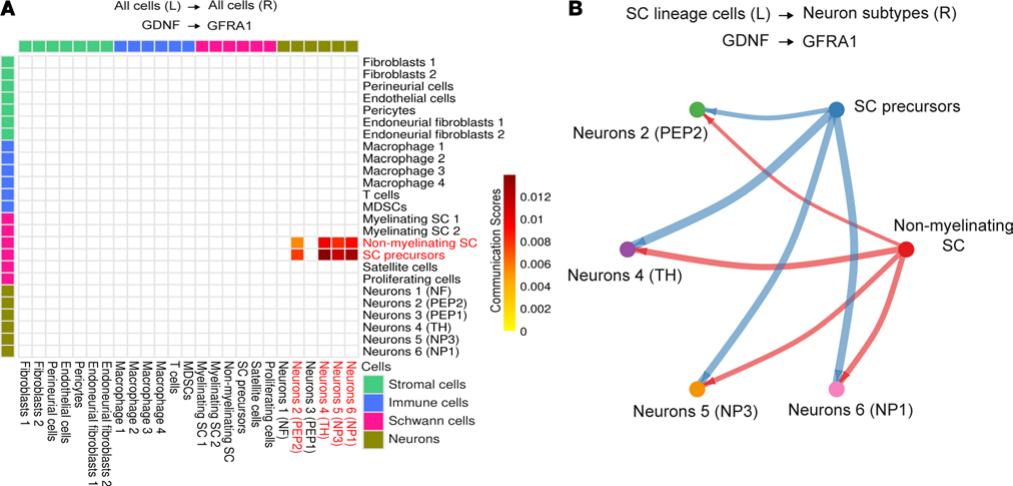
GDNF signaling from SC to neurons is enhanced in DhhCre Nf1^fl/fl^ DRGs. (**A**) Analysis of cell-cell signaling in DhhCre Nf1^fl/fl^ DRGs indicates that GDNF signals from nonmyelinating SC and SC precursors to neurons containing its co-receptor (GFRa1). (**B**) Four neuronal subtypes (TH^+^, peptidergic 2 [PEP2], nonpeptidergic 3 [NP3], and NP6) display unique signaling of GDNF with SC precursors (SCPs) and nonmyelinating SC in 7-month tumor compared with 2-month control/pretumor or 7-month control. L, ligand; R, receptor.

Increased levels of cytokines, growth factors, and other molecules have been found in neurofibromas of DhhCre Nf1^fl/fl^ mice (14) and in SCs derived from Nf1^–/–^ mice (65). Many of these molecules are known to play important roles in the modulation of pain (61, 62, 66–68). To begin to determine mechanisms through which SCs cause peripheral sensitization under normal and pathological conditions, we performed analysis of existing single-cell RNA-Seq (scRNA-Seq) data obtained from the DRGs of DhhCre Nf1^fl/fl^ mice and controls at 2 months of age (69, 70). Of the few cytokine/chemokine/growth factor transcripts that differed in SC clusters between controls and Nf1 mutants before tumor formation predicted to uniquely affect neurons, GDNF was the only factor upregulated in SCPs and in nonmyelinating SCs (Figure 5A). The CellChat algorithm also was used to predict cell types that express GDNF receptors; DRG neuron types were identified based on Usoskin et al. (71). This signaling prediction analysis indicated that enhanced SC-derived GDNF potentially targeted several sensory neuron subtypes, including the nonpeptidergic neurons (Figure 5B) that are likely those observed to be sensitized in the DhhCre Nf1^fl/fl^ mice as defined by ex vivo recording (see Figure 3). Other genes deregulated in SCs and other cell types predicted to influence neurons are shown in Supplemental Figure 5, A–C. Real-time quantitative polymerase chain reaction validated that levels of GDNF transcript (*P* < 0.05 vs. Nf1^fl/fl^ controls; 1-way ANOVA) were selectively elevated in DhhCre Nf1^fl/fl^ DRGs/nerve compared with controls (Table 2). In contrast, expression of these genes, including GDNF, was not affected by sensory neuron *Nf1* heterozygosity (Supplemental Figure 5D).

**Figure 6:**
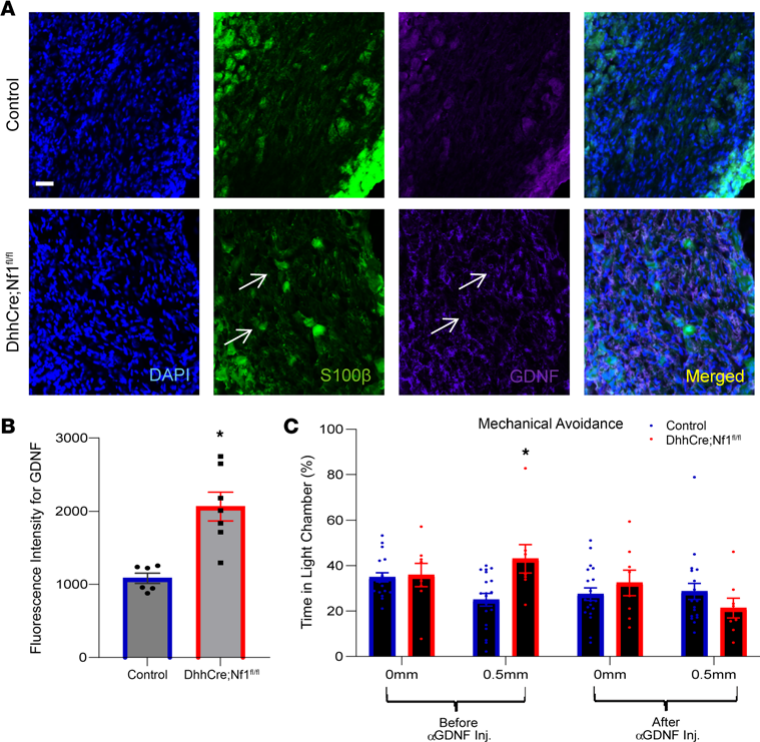
GDNF is elevated in the DRG of DhhCre Nf1^fl/fl^ when compared with control mice and regulates behavioral hypersensitivity. (**A**) Representative images of DRGs stained with different markers including S100β (green), GDNF (purple), and DAPI (blue). GDNF is mainly expressed in glial cells (arrows). Scale bar, 100 μm. (**B**) Quantifying the fluorescence intensity from each image shows elevated GDNF in DRGs of DhhCre Nf1^fl/fl^ mice compared with controls (*P < 0.05 vs. control, 1-way ANOVA with Tukey’s post hoc; mean ± SEM). (**C**) DhhCre Nf1^fl/fl^ mice normally display mechanical hypersensitivity in the MCA assay at 4 months; however, 24 hours after being injected (i.v.) with GDNF-targeting antibody, mechanical avoidance is reduced to control levels (*n* = 19 control, *n* = 8 mutant). (*P < 0.05 vs. controls, 2-way ANOVA, with Tukey’s post hoc; mean ± SEM.)

We then verified the increased expression of GDNF in SCs of the DRG and quantified subpopulations of sensory neurons using immunohistochemical analysis. We found a significant increase in GDNF in S100β^+^ satellite glial cells and SC/nerve roots in DhhCre Nf1^fl/fl^ mice compared with Cre-negative controls (Figure 6, A and B). No changes in the neuronal markers TRPV1, IB4, or ASIC3, which mark distinct subpopulations of sensory neurons, were found in the DRGs of DhhCre Nf1^fl/fl^ mice compared to controls (Supplemental Figure 6, A and B). To determine if SC- produced GDNF plays a role in the hypersensitivity in the NF1 mouse model, we treated DhhCre Nf1^fl/fl^ mice in vivo with a GDNF-targeting antibody and performed MCA analysis. The treatment rescued the mechanical hypersensitivity that is normally observed in DhhCre Nf1^fl/fl^ animals for at least 48 hours (Figure 6C and Supplemental Figure 6).

## Discussion

Our data validate an important role for SCs in nociceptive processing. The targeted knockout of the *Nf1* gene in SC/SCPs (but not sensory neurons), prior to tumor formation, in a genetically engineered mouse model of NF1 (40) caused increased hypersensitivity at the afferent and behavioral levels (Figures 1 and 2). Similar results were obtained by chemogenetically increasing SC calcium in WT mice (Figure 3). Blocking enhanced SC calcium in the DhhCre Nf1^fl/fl^ mice using inhibitory DREADDs blunted the observed mechanical hypersensitivity (Figure 4). These gain- and loss-of-function experiments strongly support the idea that calcium-mediated effects in SCs contribute to hypersensitivity. Of the factors upregulated in DRGs by DREADD-dependent calcium increases in SC/SCPs (Figure 3), and by specific deletion of *Nf1* in SCs, we identified induction of GDNF expression. This corresponded with enhancement of predicted GDNF signaling to neurons from SCs as assessed by scRNA-Seq analysis (Figure 5). Finally, targeting GDNF with systemic antibody treatment reduced mechanical hypersensitivity in the NF1 mouse model (Figure 6).

Glial cells play a pivotal role in the functioning of the nervous system. Multiple roles from regulating neuronal survival and differentiation during embryogenesis (72), to modulating the formation of myelin sheaths, maintaining the appropriate concentrations of ions in the nerve milieu, and regulating nociception are known (5, 6, 8, 72, 73). In the periphery, SCs are known to provide an early response to nerve injury and to initiate repair and facilitate axon regeneration (74). Recently, SCs have been shown to also play a pivotal role in the development and maintenance of pain by proliferating and interacting with nociceptive neurons to release factors such as chemokines/cytokines/growth factors (5, 7, 75, 76). As recent studies have been focused on neuron-glia crosstalk, strategies targeting this interaction have gained traction as potential therapies for pain.

Here we found that DREADD-dependent activation of SCs, which increased SC calcium signaling, was sufficient to induce mechanical hypersensitivity in adjacent sensory neurons. This afferent sensitization likely underlies the behavioral hypersensitivity found in these transgenic mice (Figure 3) (4, 45, 77–79). We also found that chemogenetic activation of SCs upregulates a specific set of growth factors and cytokines that may influence sensory function (Table 1). There are, of course, a number of additional ways that Gq signaling in SCs could alter somatosensory processing. These include regulation of ion channels that can modulate the electrochemical gradient in the nerve and/or release of other factors that may modify structural integrity of the nerve (80–83). However, concurrent regulation of cAMP also occurs through Gi/Gq as does prolongation of RAS effects through annexins. Involvement of each of these pathways will need to be explored in future studies. Given the key role of Ca2^+^ release from cells, here we focused on potential calcium-mediated effects.

It is important to note that in order to observe an effect of DREADD-dependent activation or inhibition of peripheral SCs (Figures 3 and 4), 7 days of CNO/C21 were required. This may indicate that persistent activation of SCs is required for factors to be produced in sufficient quantities to affect adjacent sensory neurons in the PNS so that a behavioral effect is noted along with afferent sensitization. Activation of a DREADD by CNO (or C21) is transient and typically only lasts up to approximately 2 hours in vivo (see ref. 57). We delivered a single dose daily; therefore, more frequent administration is required to alter SCs and subsequent behavior.

Prior studies also did not address the timing of pain onset in Nf1 animal models (22, 27, 35, 36). We therefore utilized several transgenic lines to define cell types involved in hypersensitivity upon *Nf1* mutation. Previous work on NF1-related pain has focused on use of haploinsufficient mice (Nf1^+/–^), edited *Nf1* in adult animals using guide RNAs, or studied the release of neuropeptides from sensory neurons of Nf1^+/–^ animals under injury conditions (22, 84). These in vivo studies have provided some information on how pain may develop in NF1, but they do not provide understanding of how specific cell types contribute to the onset of NF1-associated pain. In haploinsufficient mice and after gene editing, multiple nerve cell types are affected. Studies using dissociated neurons in vitro have suggested that these cells can display enhanced excitability upon *Nf1* mutation (85), but in vivo, an optimized environment may be necessary to observe sensitization (Figures 1 and 2).

A controversy in the field is whether mouse models of NF1 actually show a pain-related phenotype without secondary injury to the peripheral nerves. In our studies, the commonly used model of NF1 (e.g., Nf1^+/–^ mice) does not show a pain-like phenotype when using standard evoked assessments of pain, such as Randall-Selitto testing (Figure 1) or the related von Frey withdrawal response, consistent with previous work (35). Similar to the Nf1^+/–^ mouse, sensory neuron *Nf1* mutants and SC/SCP *Nf1* mutants display minimal effects using Randall-Selitto testing (Figure 1). In tests that provide a choice for the animal, such as the MCA, Nf1^+/–^ mice reveal mechanical hypersensitivity. MCA analysis also reveals a role for SC/SCP *Nf1* in pain-like behaviors that is not observed in the sensory neuron mutants (Figure 1). This indicates that assays that allow the animal to choose between stimuli are sensitive indicators of pain in models of NF1. Interestingly, mechanical hypersensitivity was observed most often at the smallest spike height (0.5 mm) in DhhCre Nf1^fl/fl^ mice (Figure 1). The basis for this would need to be more directly assessed in future studies.

In the DhhCre Nf1^fl/fl^ preclinical model of NF1, tumors form in the cervical region around 4 months of age. Small tumors can form in the lumbar DRG, which innervate the hind limb; however, tumors are not visible until 6–9 months of age. Tumors are preceded by disruptions in nerve structural integrity that are also present in human nerve and tumors (40, 56). Although we cannot rule out a role for Remak bundle disruption (Supplemental Figure 1) in pain-related behaviors in the DhhCre Nf1^fl/fl^ mice, since the axons are directly exposed to the extracellular environment in the nerve, results from the Gq DREADD experiments (Figure 3) indicate that changes in SCs alone are capable of inducing hypersensitivity. Future experiments will be needed to address how or if Remak bundle disruption contributes to pain in NF1.

In our choice assay, DhhCre Nf1^fl/fl^ mice with chemogenetic inhibition of SC calcium (e.g., suppression of the enhanced calcium found in Nf1^–/–^ SCs) (56) reversed observed mechanical hypersensitivity (Figure 4). However, it was important in this assay to eliminate light as an aversive stimulus (Supplemental Figure 4). Previous reports suggest that other *Nf1*-mutant mice also display enhanced light sensitivity (86). Chemogenetic inhibition of calcium in SC/SCPs mutant for *Nf1* appeared to have resulted in a loss of light aversion. It will be important in the future to assess light sensitivity in diverse mouse models of NF1 to identify the cause of the phenotype. Another point to note is that the Gi DREADD affects cAMP signaling and potassium efflux in addition to calcium (57). Changes in cAMP in SCs have been shown to evoke sustained mechanical allodynia in a mouse model of migraine pain elicited by calcitonin gene-related peptide (CGRP) (87). Although we did not test for CGRP signaling, it will be necessary in future experiments to determine the potential roles, if any, for CGRP and other factors in NF1-related hypersensitivity. In spite of these limitations, our data indicate that prior to tumor formation, mutations in SC/SCP *Nf1* are key players in pain-like behaviors. This finding supports clinical reports that individuals with NF1 often report pain in parts of the body that are not obviously affected by tumors (21).

SC/SCP deletion of *Nf1* induced robust mechanical sensitization in HTMRs and CPM neurons that could underlie behavioral hypersensitivity (Figure 2). Intriguingly, heat hypersensitivity is not observed, consistent with some models of NF1 as well as patient reports of a lack of heat-related pain (35). Our results are not consistent with recent reports in which intrathecal injections of guide RNAs targeted *Nf1* in adult rats (22), possibly because that deletion was targeted to the adult nervous system and not to SCs (40). Our results are also inconsistent with reports that show heat hypersensitivity in the Nf1^+/–^ mouse after injury (15). Enhanced heat hypersensitivity might be observed if the environment is optimized, for example when immune cells are recruited to the nerve after injury. This is consistent with the increase in tumor formation in NF1 mice after nerve injury (88, 89).

SCs can modulate nociception by releasing factors including chemokines, growth factors, and cytokines (7, 11, 43, 60, 61, 68, 90–92). Neurotrophic factors enable neuronal outgrowth, and alterations in levels of these factors can also influence peripheral sensitization (43, 52, 93, 94). An intriguing finding in our study is that there is no increase in cytokines/growth factors in the DRGs from mice with sensory neuron *Nf1* knockout (PirtCre Nf1^+/fl^) (Supplemental Figure 5D). Rather, GDNF is elevated uniquely in the SC/SCP *Nf1* knockout (Table 2). Further, pathway analyses using scRNA-Seq data from DhhCre Nf1^fl/fl^ mice prior to any tumor formation, at 2 months of age, indicate that of all signaling pathways that are predicted to be increased in SCs for communication with neurons, GDNF signaling is the only one specifically elevated (Figure 5). GDNF and the related GDNF family factor, artemin, have been linked to afferent sensitization and pain in animal models and in clinical studies, and targeting this signaling molecule has gained interest as a therapeutic strategy for pain (93, 95). After validating GDNF expression in glial cells of the DhhCre Nf1^fl/fl^ PNS (Figure 6), we found that treatment of DhhCre Nf1^fl/fl^ mice with GDNF-targeting antibodies suppressed noxious mechanical avoidance in the MCA assay for at least 48 hours (Figure 6). Although SCs may not be the sole source of GDNF (90, 96), this result strongly supports a major role of SCs in modulating afferent sensitization in NF1. This concept is further supported by our finding that nonpeptidergic CPM neurons are predicted to be affected by GDNF in the DhhCre Nf1^fl/fl^ mice. These cells are known to be IB4^+^ and GFRa1^+^ (97) and directly respond to GDNF. Together, our findings contribute to the increasing evidence implicating interactions between non-neuronal cells and sensory neurons in effects on nociception and extend it by application to NF1.

Pain can substantially impede daily activities in patients with NF1, yet treatment for pain in NF1 remains a major a challenge for clinicians (22, 35–37). SC/SCPs have been well established to play an important role in tumor formation (14, 40, 65), and our data suggest that they also play a key role in pain-like behavior, independent of tumors. This study also suggests what we believe to be a unique approach to treat pain in NF1, by blockade of GDNF.

## Methods

### Animals.

Male and female mice between 1 and 7 months of age were used in all studies. All transgenic mice used in these studies were bred in house. Mice expressing a Gq-coupled DREADD specifically in SCs were used in initial experiments. To generate this mouse, we used the Dhh-Cre mouse, which expresses Cre recombinase in SCs and SCP. This line was crossed to a Cre-dependent Gq-coupled DREADD mouse (Rosa26-LSL-hM3Dq) (Jackson Laboratory) to obtain a line that allows for DREADD-dependent modulation of SC activity. In other studies, to knock out *Nf1* in SCs and SCP, we crossed the Dhh-Cre mouse to a Nf1^fl/fl^ (Jackson Laboratory) line to create the DhhCre Nf1^fl/fl^ mouse model of NF1 (40). Similarly, to target deletion of *Nf1* to sensory neurons, we utilized the PirtCre mouse (donated by Xinzhong Dong, Johns Hopkins University, Baltimore, Maryland, USA), which targets Cre recombinase expression in sensory neurons (98), and crossed it with the Nf1^fl/fl^ mice. Nf1^+/–^ haploinsufficient mice (35) and mice with SC and sensory neuron heterozygous mutations in *Nf1* (DhhCre PirtCre Nf1^+/fl^) were used for comparisons. Additional experiments were performed as indicated on DhhCre Nf1^fl/fl^ mice that contained a Cre-dependent Gi-coupled DREADD (hM4Di) (Jackson Laboratory) in SCs. Mice were housed in a barrier facility, were maintained on a 14-hour light/10-hour dark cycle with a temperature-controlled environment, and were given food and water ad libitum.

### Treatments.

Mice were treated with DREADD agonist CNO at 2 mg/kg/d for 1–7 days or C21 at 20 μg/μL/d for 1–7 days (TOCRIS) in vivo along with their littermate controls. In other experiments, mice were injected with GDNF-targeting antibody intravenously at 5 μg/g (ANT-014, Alomone) in vivo along with their littermate controls. For dissociated SC experiments in vitro, cells were treated with CNO at 10–40 μM alone or C21 at 20–200 μM with or without 100 μM of ATP. The doses for ATP to induce calcium fluorescence and CNO or C21 to suppress the fluorescence were determined using a Synergy H1 plate reader (BioTek, Agilent). Then the separate cultures were treated with vehicle, ATP, and vehicle + ATP in comparison with ATP + CNO and ATP + C21 at the newly determined doses. This was performed in triplicate (*n* = 68–164 cells per well), and all images were captured on a Nikon A1R inverted fluorescence microscope and intensity measurements obtained using Nikon NIS-Elements 2 software.

### Pain-related behaviors.

All behavioral analyses were performed by experimenters following a protocol blinded to genotype/treatment. To assess evoked hypersensitivity, nociceptive withdrawal thresholds were determined using a Randall-Selitto apparatus (IITC Life Science). Before the test, the animal was acclimatized in the behavior room for 25–30 minutes. The animal was scuffed, then carefully immobilized, and the right paw was placed on the platform with an application of an increasing mechanical force, in which the tip of the device was applied onto the medial portion of the hairy skin surface of the hind paw until a withdrawal response was observed. The maximum force applied was limited to 250 g to avoid skin damage. The test was repeated 3 times with a 5-minute interval between stimuli (48). The average of the 3 trials was determined per mouse, and data were averaged per condition for comparisons.

To assess the animals’ choice to avoid either an aversive light stimulus or a noxious mechanical stimulus, the MCA assay was used (99). Mice were placed in a chamber for a brief period (~10 seconds), and then a bright light was illuminated. A door to escape the light chamber was then opened to allow free access to a darker chamber after crossing through a small middle tunnel with a floor that contained varying levels of metal spikes. Mice were allowed to complete the task 4 times for a duration of 3 minutes each. On each trial, the floor of the middle chamber was raised from 0 mm to 2 mm in 0.5 mm increments. The 0.5 mm spike height was the smallest size while 2 mm was the highest spike height used. Time spent in each chamber was recorded, and percentage time avoiding the light or mechanical stimulus was determined per mouse and then averaged per group for comparison.

For the choice assay (no light), mice were placed in a 3-chamber setup for 3 minutes for acclimatization. The first chamber was empty, the second chamber contained the varying levels of spikes similar to that described for the MCA, and the third chamber contained bedding from the housing where the mouse resided. For the experiment, the mouse was placed in first chamber for 10 seconds. A door to escape the first chamber was then opened to allow free access to a bedding chamber, which was provided after crossing through a second chamber that contained varying levels of metal spikes. Mice were allowed to complete the task 4 times for a duration of 3 minutes each. On each trial, the floor of the middle chamber was raised from 0 mm to 2 mm in 0.5 mm increments. Time spent in each chamber was recorded, and the percentage time avoiding the first chamber that was devoid of bedding was used for comparison with the control. All behavioral assessments were performed in our groups at 1–2 months, 4–5 months, and/or 7–9 months of age.

### Ex vivo recording preparation.

The ex vivo hairy hind paw skin/saphenous nerve/DRG/SC somatosensory system recording preparation was performed as described previously (53). The intracellular single-unit recordings were performed on the L2/L3 DRGs using the quartz microelectrode containing 5% Neurobiotin (Vector Laboratories) in 1 M potassium acetate. Electrical stimuli were delivered through a suction electrode from the nerve to identify sensory neuron somata with axons contained in the saphenous nerve. When the cell was found to be electrically driven, the peripheral RF was localized using a small paintbrush or hot (~51°C) or cold (~1°C) physiological saline if no mechanical RF was found. Once identified, RFs were then probed with an increasing series of von Frey filaments (0.07–10 g, if mechanically sensitive) for 1–2 seconds to assess mechanical responsiveness.

After mechanical responsiveness was determined, a controlled thermal stimulus was applied using a 3 × 5 mm contact area Peltier element (Yale University Machine Shop). Cold stimuli consisted of a variable-rate cold ramp beginning at 31°C, dropping to approximately 2°C to 4°C, holding for 4 to 5 seconds, and slowly returning to 31°C. After bath temperature was maintained for approximately 4 to 5 seconds, a heat ramp was applied, which went from 31°C to 52°C in 12 seconds. This heat stimulus was then held at 52°C for 5 seconds. The stimulus then ramped back down to 31°C in 12 seconds. Adequate recovery times (approximately 20–30 seconds) were employed between stimulations. All elicited responses were recorded digitally for offline analysis of thresholds, firing rates, and mean peak instantaneous frequencies to the various stimuli using Spike2 software (Cambridge Electronic Design).

### Immunohistochemistry.

DRGs from mice, at the indicated time points, were removed and immersion-fixed in 3% paraformaldehyde in 0.1 M phosphate buffer (PB) for 30 minutes at room temperature. Fixed DRGs were embedded in OCT (Sakura Finetek USA, Inc.) and incubated at –80°C. DRG sections were cut on a cryostat at 12 μm and mounted onto the slides. Sections were then fixed for about 15 minutes, blocked, and incubated overnight with up to 2 of the following primary antibodies: transient receptor potential vanilloid type 1 (rabbit anti-TRPV1, Alomone; 1:3,000), acid-sensing ion channel 3 (guinea pig anti-ASIC3, MilliporeSigma; 1:2,000), S100β (rabbit anti-S100β, Abcam 1:1,000), or GDNF (rabbit anti-GDNF, Abcam 1:500). Sections were then incubated with appropriate fluorescently conjugated secondary antibodies (Jackson ImmunoResearch anti–guinea pig Alexa Fluor 647, 1:400; or Jackson ImmunoResearch anti-rabbit Alexa Fluor 594, 1:400). Slides were coverslipped in Fluro-Gel (Electron Microscopy Sciences) and stored in the dark at room temperature until imaged. In other cases, after fixation, DRGs were embedded in 10% gelatin followed by immersion in 10% sucrose in 0.1 M PBS overnight. DRG sections were cut on a sliding microtome (Microm HM 430, Thermo Fisher Scientific) at 50 μm and placed in 12-well plates containing 0.1 M PBS, pH 7.3. Sections were then blocked for 1.5 hours and incubated overnight with GFP (anti-GFP in chicken, Abcam, ab13970; 1:1,000) and NeuN (anti-NeuN in rabbit, Abcam, ab177487; 1:500) primary antibodies. Sections were then washed and incubated with appropriate fluorescently conjugated secondary antibodies (FITC donkey anti-chicken, Abcam, ab63507, and Jackson ImmunoResearch donkey anti-rabbit, 711-605-152; 1:400) for 1.5 hours at room temperature. Sections were then placed on gelatin-coated slides prior to coverslipping. Labeling was characterized and documented using a Nikon confocal microscope with sequential scanning to avoid bleed-through of the different fluorophores. For quantification, 3 images were taken from the 3 different slides from 3 different animals along with their respective controls. The final intensity was used to generate the graphs as shown in the Results section.

### Electron microscopy.

Mice used for electron microscopy were perfusion-fixed in a solution combined with 4% paraformaldehyde and 2.5% glutaraldehyde in 0.1 M PB at pH 7.4. The saphenous nerve was dissected out, postfixed in the same fixation overnight, then transferred to 0.175 mol/L cacodylate buffer, osmicated, dehydrated, and embedded in Embed 812 (Ladd Research Industries). Semithin sections were cut, and the best block was selected for ultrathin sections. Ultrathin sections were stained in uranyl acetate and lead citrate and viewed on a Hitachi H-7600 microscope. Remak bundles were counted from the photographs and grouped into 1–2, 3–5, and more than 6 Remak bundles, and percentage of Remak bundles was calculated and compared between genotypes.

### scRNA-Seq.

CellChat (http://www.cellchat.org, version 1.6.0) objects were created from 4 Seurat (https://satijalab.org/seurat, version 3.1.2) objects — 2- and 7-month-old WT mouse DRG controls, 2-month-old mouse neurofibroma pretumor (DhhCre Nf1^fl/fl^), and 7-month-old mouse neurofibroma tumor (DhhCre Nf1^fl/fl^) — extracted from the 10× scRNA-Seq data (70).

The “secreted signaling interactions” subdatabase (for mouse) was chosen to infer the cell state–specific communications. Briefly, CellChat identifies overexpressed ligands or receptors in one cell group and then identifies overexpressed ligand-receptor interactions if either ligand or receptor is overexpressed. CellChat infers the biologically significant cell-cell communication by assigning each interaction with a probability value and performing a permutation test. These steps create complex cell-cell communication networks with assigned communication probability scores.

After inferring aggregated cell-cell networks, we removed autocrine interactions and focused on cell-cell interactions where SC lineage cells and neuron subtypes participate as sources (i.e., ligand-expressing) and targets (i.e., receptor-expressing), respectively. The *P* value of 0.05 was chosen to extract significant ligand-receptor (LR) interactions for each sample set. We investigated (a) 2-month pretumor (case) versus 2-month control, (b) 7-month control (case) versus 2-month control, (c) 7-month tumor (case) versus 2-month pretumor, and (d) 7-month tumor (case) versus 7-month control and extracted unique LR pairs only detected in the case sample from each comparison. These unique LR pairs were visualized using circle plots, including SC lineage and neuron subtypes. The same LR pairs were searched against all cell types and visualized using heatmaps (Figure 4). Neuron subtypes were re-annotated based on a previous report (69): Neuron 1 = low-threshold mechanoreceptors (NF), Neuron 2 = lightly myelinated Aδ nociceptors (PEP2), Neuron 3 = C-type thermo-nociceptors (PEP1), Neuron 4 = C-low-threshold mechanoreceptors (TH), Neuron 5 = itch-specific sensory neurons (NP2/3), and Neuron 6 = polymodal nociceptors (NP1).

### Real-time PCR.

DRGs (L2/3) were collected, and RNA was isolated using a QIAGEN RNeasy Mini Kit according to the manufacturer’s protocol. We reverse-transcribed 1 mg samples of purified RNA into cDNA with M-MLV Reverse Transcriptase (Promega). Then 25 ng samples of cDNA were used in SYBR Green real-time PCR reactions on a Step-One real-time PCR machine (Applied Biosystems). Cycle threshold (Ct) values for all targets were all normalized to a GAPDH internal control, and fold-change was determined as 2^ΔΔCt^ (Applied Biosystems). Values were converted and reported as a percentage change, where 2-fold change = 100% change. Primers used are listed in Supplemental Table 1.

### Statistics.

All data sets were analyzed using GraphPad Prism statistical software. Data are represented as mean ± SEM. Significance was defined as *P* ≤ 0.05. For behavioral analyses, 1- or 2-way ANOVA with Tukey’s post hoc test were performed. For electrophysiological analyses, 1- or 2-way ANOVA on ranks with Dunn’s post hoc test were performed. For PCR, 1-way ANOVA with Tukey’s test were performed. For cell culture and immunohistochemistry, 2-tailed *t* test or 1- or 2-way ANOVA with Tukey’s test were performed. Statistical values are listed for each figure in Supplemental Table 3.

### Study approval.

All procedures were approved by the Cincinnati Children’s Hospital Institutional Animal Care and Use Committee and adhered to the *Guide for the Care and Use of Laboratory Animals* (National Academies Press, 2011) under the Association for Assessment and Accreditation of Laboratory Animal Care International–approved practices.

### Data availability.

All data are provided in the Supporting Data Values XLS file or can be provided by the corresponding author upon written request. Sequence data are available with NCBI GEO accession GSE181985.

## Author contributions

NGRR contributed to experimental design, conducted the majority of the experiments, performed data analysis, and prepared and edited the manuscript. LAM performed ex vivo recording experiments on DhhCre hM3Dq mice. LMO performed PCR and behavioral analyses on select cohorts. IM performed PCR, ex vivo recording, and some behavioral tests. AA contributed to conducting cell culture experiments, data analysis and interpretation for ex vivo recordings, and treatment with DREADD agonist (C21). KLS contributed to IHC and PCR experiments. ARR performed additional IHC and PCR tests along with data analysis. LB performed supplementary MCA assays and PCR. MCH performed all genotyping and animal husbandry. JPC contributed to cell culture experiments and confocal imaging. TAR performed electron microscopy (EM) and data analysis. LFQ contributed to experimental design and performed behavioral and electrophysiological tests along with manuscript editing. KC performed scRNA-Seq, data analyses, and figure generation. NR contributed to experimental design, provided transgenic animals, oversaw EM studies and scRNA-Seq analyses, and edited the manuscript. MPJ contributed to experimental design, performed ex vivo recordings, contributed to confocal imaging, analyzed and interpreted the data, and prepared the manuscript.

## Supplementary Material

Supplemental data

Supporting data values

## Figures and Tables

**Table 1:**
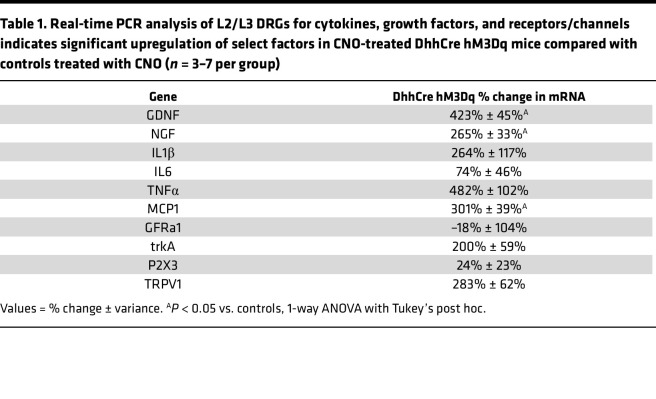
Real-time PCR analysis of L2/L3 DRGs for cytokines, growth factors, and receptors/channels indicates significant upregulation of select factors in CNO-treated DhhCre hM3Dq mice compared with controls treated with CNO (*n* = 3–7 per group)

**Table 2:**
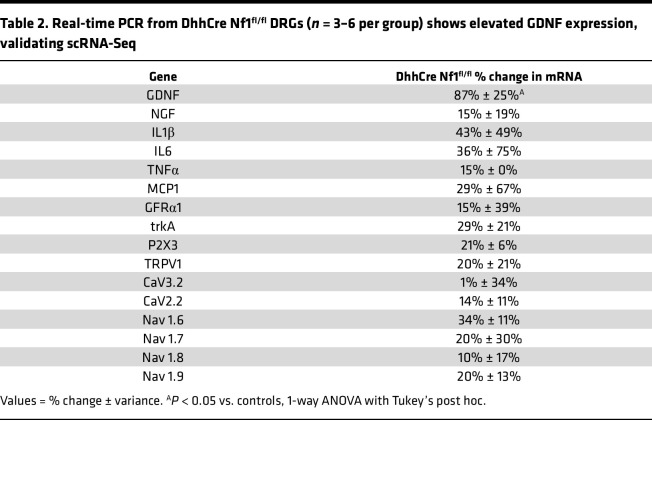
Real-time PCR from DhhCre Nf1^fl/fl^ DRGs (*n* = 3–6 per group) shows elevated GDNF expression, validating scRNA-Seq

## Version 1 01/23/2024

Electronic publication
