# Supplementary material for: Schwann cells modulate nociception in neurofibromatosis 1
Source: JCI Insight. 2024 Jan 23;9(2):e171275. doi: 10.1172/jci.insight.171275 (PMC10906222; doi:10.1172/jci.insight.171275)
Supplement: Supplemental data [file jciinsight-9-171275-s116.pdf]

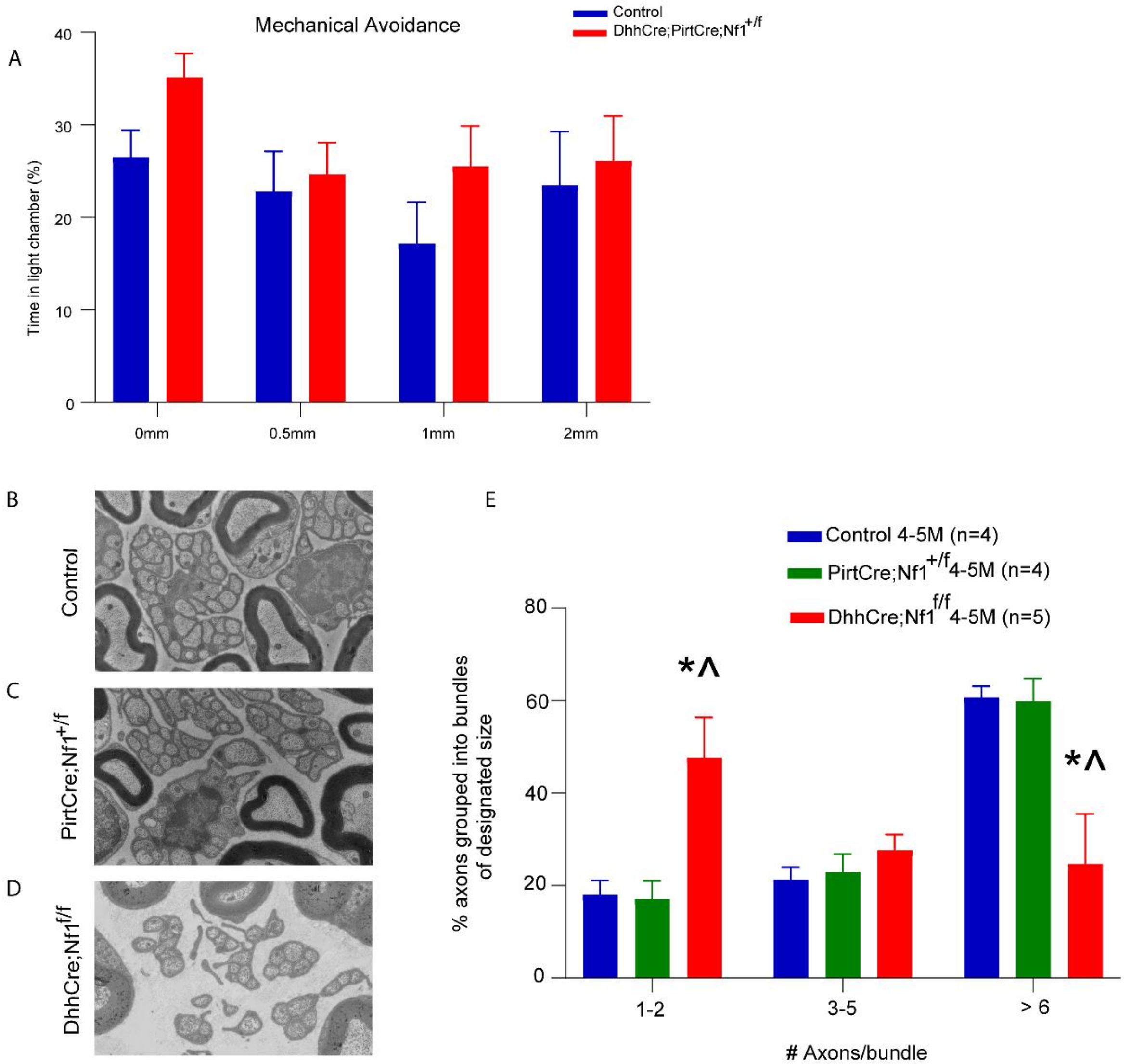

Supplementary Figure 1: Behavioral analysis of mice with one copy of *Nf1* deleted from both SCs and sensory neurons. A: No differences in mechanical avoidance are found in mice with one copy deleted from SCs and sensory neurons (DhhCre;PirtCre;Nf1$^{+/f}$) compared to controls (control= 14, mutant= 14, $p > 0.05$, 2-way ANOVA, Tukey's post hoc; Mean ± SEM). **B**: Electron micrograph of the saphenous nerve shows intact Remak bundles surrounding the axons in Nf1+/- mice at 4-5 months (Control). **C**: Representative electron micrograph of saphenous nerves in PirtCre;Nf1$^{+/f}$ shows intact Remak bundles at 4-5

months. **D**: However, significant Remak bundle disruption is observed in the saphenous nerve of 4-5-month-old DhhCre;Nf1$^{f/f}$ mice. **E**: Quantification of percentage of axons grouped within a Remak bundle in the groups (*p<0.05 vs control; ^p<0.05 vs PirtCre;Nf$^{+/f}$, 2-way ANOVA with Tukey's post hoc; Mean ± SEM).

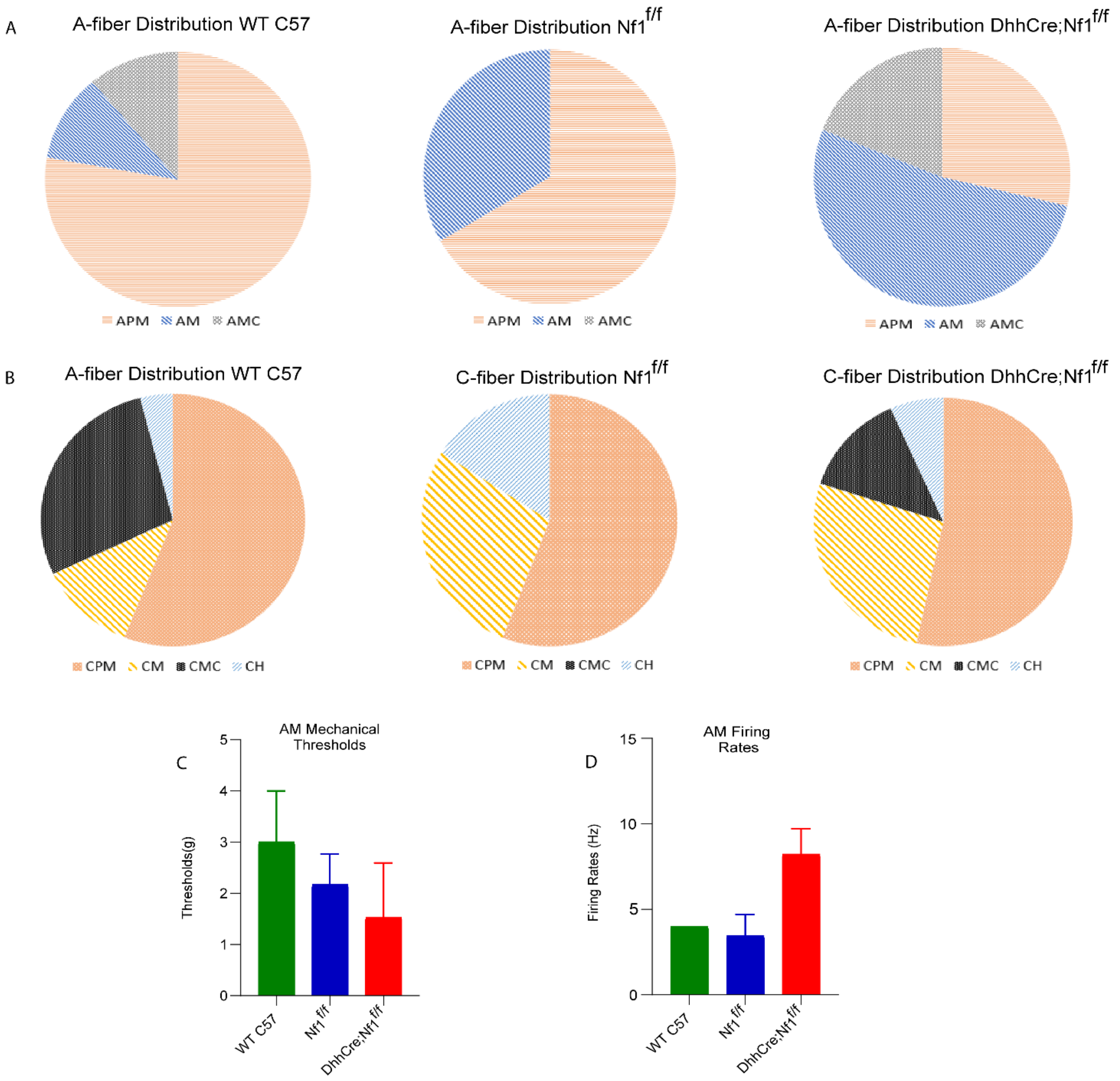

Supplementary Figure 2: Response properties and distribution of A-fibers and C-fibers in WT C57Bl6, Nf1$^{f/f}$ and DhhCre;Nf1$^{f/f}$ mice.

A: Distribution of A-fibers in C57Bl6, Nf1$^{f/f}$ and DhhCre;Nf1$^{f/f}$ mice: APM: A-polymodal, AM: mechanosensitive only, AMC: A-mechano-cold (n= 14 APM, 2 M, and 2 AMC for C57BI6, 12 APM, and 6 AM for Nf1$^{f/f}$; n= 4 APM, 8 AM and 3 AMC for DhhCre;Nf1$^{f/f}$). **B:** Distribution of C-fibers in our three groups. CPM: C-polymodal, CM: C-mechano, CMC: C-mechano cold,

CH: C-heat (n= 14 CPM, 3 CM, 7 CMC, and 1 CH for C57Bl6; n= 14 CPM, 7 CM, and 4 CH for $Nf1^{f/f}$; n= 5 CPM, 4 CM, and 2 CMC DhhCre;$Nf1^{f/f}$). **C-D**: The mechanical thresholds and firing rates of AM fibers were not different in DhhCre;$Nf1^{f/f}$ mice compared to controls (C57Bl6 WT= 2, $Nf1^{f/f=}$6 and DhhCre;$Nf1^{f/f}$=11, p>0.05, 2-way ANOVA, with Tukey's post hoc; Mean ± SEM).

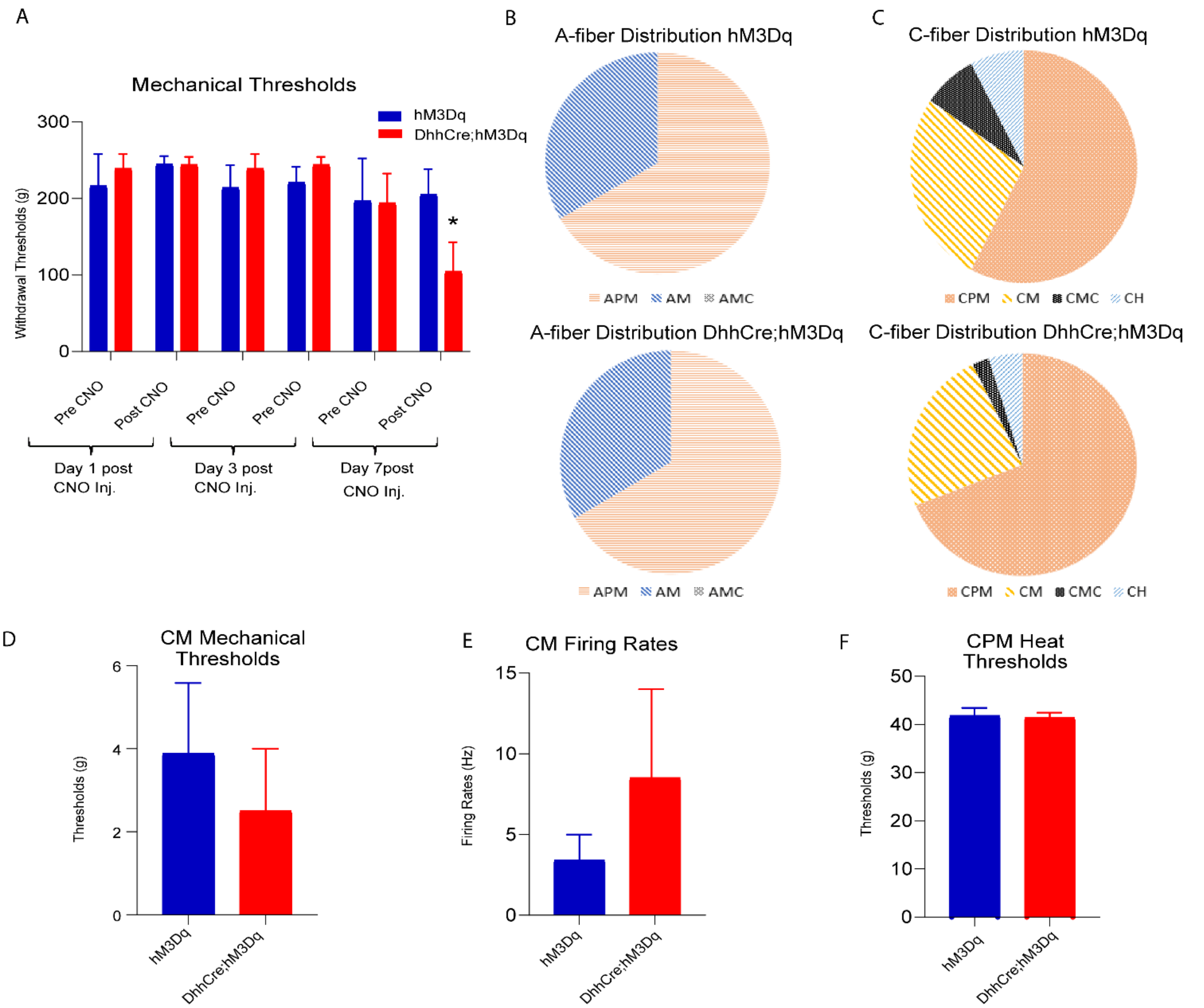

Supplementary Figure 3: CNO dose response analysis and additional response properties obtained from DhhCre;hM3Dq mice. **A:** Injection of CNO for seven days induces peripheral hypersensitivity in hM3Dq mice while no change in the mechanical hypersensitivity is observed after 1 or 3d of CNO (n=5 control, n=5 mutant)/ *p<0.05, 1-way ANOVA with Tukey's post hoc). **B:** Distribution of various subtypes of A-fibers found in hM3Dq and DhhCre;hM3Dq mice during *ex vivo* recording; APM: A-polymodal, AM: mechanosensitive only, AMC: A-mechano-cold (n= 2 APM, and 1 AM for control; 2 APM, and 1 AM for mutant). **C** : Distribution of C-fibers in DhhCre;hM3Dq mice compared to controls

(hM3Dq); CM: C-mechano, CMC: C-mechano cold, CH: C-heat (n= 7 CM, 2 CMC and 2 CH for control; n= 10 CM, 1 CMC and 2 CH for mutant). **D-E**: The mechanical thresholds and firing rates of CM fibers were not different when compared to the controls (n= 7 hM3Dq, n=9 DhhCre;hM3Dq, p> 0.05vs hM3Dq, 2-way ANOVA with Tukey's post hoc; Mean ± SEM). **F**: CPM heat thresholds were also not different in DhhCre;hM3Dq mice compared to controls (n=7 hM3Dq, n=9 DhhCre;hM3Dq, p>0.05, 1-way ANOVA).

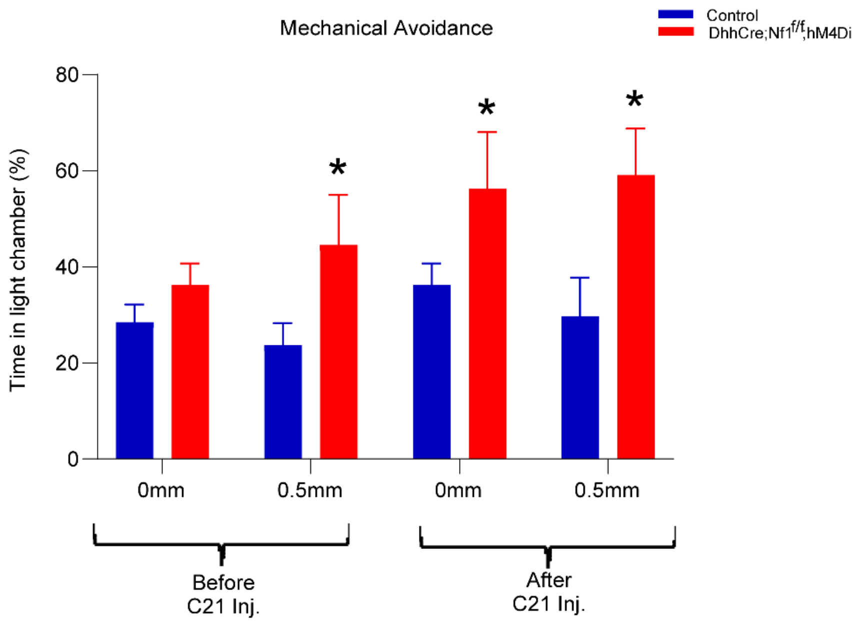

Supplementary Figure 4: Mechanical conflict avoidance assay in DhhCre;Nf1$^{f/f}$;hM4Di mice. Prior to C21 treatment, DhhCre;Nf1$^{f/f}$;hM4Di mice display expected mechanical avoidance, however after 7d of C21 treatment, mice show no avoidance of light and spend approximately equal time in the light and dark chambers regardless of whether the noxious mechanical stimulus is present. (control= 13, mutant= 10, *p<0.05 vs control, 1-way ANOVA with Tukey's post hoc test; Mean ± SEM).

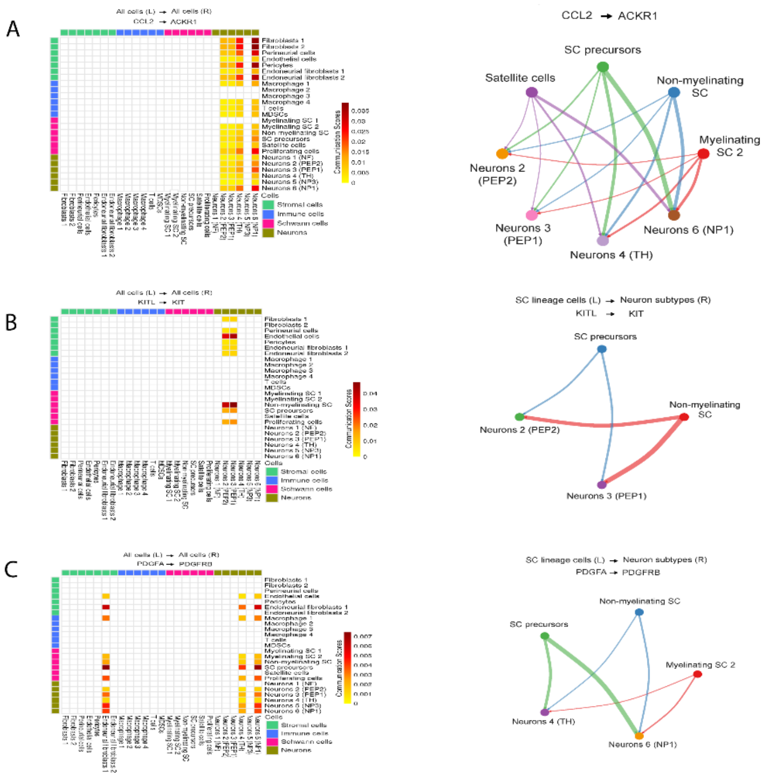

D

% change in mRNA from Control	
**Gene**	**PirtCre;Nf1(+/f)**
GDNF	-90 ± 105%
NGF	-72 ± 43%
IL1β	81 ± 54%
IL6	46 ± 33%
TNFα	-93 ± 89%
MCP1	-70 ± 44%
GFRα1	-74 ± 61%
TrkA	62 ± 14%
P2X3	56 ± 21%
TRPV1	54 ± 33%

Supplementary Figure 5: CellChat analysis of additional genes with signaling between glia and sensory neuron subtypes. The heatmaps and signaling diagrams depict CCL2-ACKR1 **(A)**, KITL-KIT **(B)**, and PDGFA- PDGFRB **(C)** interactions among multiple cell types. These plots suggest that non-myelinating SC, SC precursors, and 4 neuron subtypes display unique interaction partners in 7 mo tumor compared to 2 mo control/pretumor or 7 mo control. **D:** Realtime PCR from PirtCre;Nf1$^{+/f}$ DRGs (n=4-8 per group) shows no differences compared to controls (1-way ANOVA with Tukey's post hoc, Values = % change ± variance). L = ligand, R = receptor.

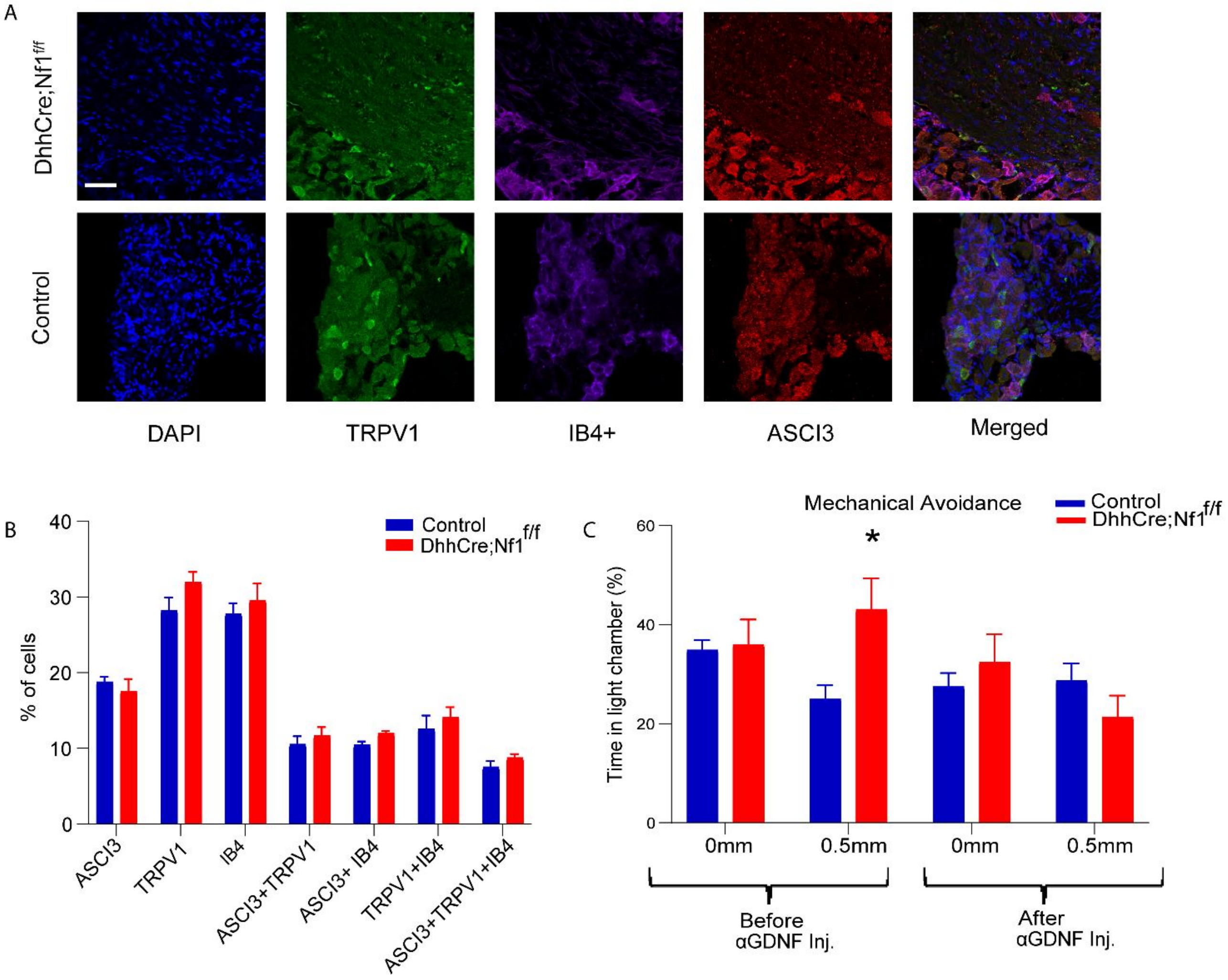

Supplementary Figure 6: Immunohistochemical analysis of DRGs for various neuronal markers and MCA data from DhhCre;Nf1$^{f/f}$ mice treated with GDNF targeting antibody at 48hr post injection. A: Examples of DRGs from control or DhhCre;Nf1$^{f/f}$ mice stained for TRPV1, IB4 or ASIC3. **B:** Quantification of numbers of cells positive for each marker. No differences in numbers of TRPV1, IB4 or ASIC3 cells are found in DhhCre;Nf1$^{f/f}$ mice compared to controls ($p>0.05$, 1-way ANOVA, with Tukey's post hoc; Mean ± SEM). **C:** GDNF targeting antibody inhibited mechanical hypersensitivity observed in DhhCre;Nf1$^{f/f}$ mice for at least 48hrs. (n=19 control; n=8 mutant, *$p>0.05$, 2-way ANOVA, with Tukey's post hoc; Mean ± SEM) scale bar= 100µm.

**Gene**	**Forward**	**Reverse**
GAPDH	ATGTGTCCGTCGTGGATCTGA	ATGCCTGCTTCACCACCTTCTT
GDNF	AGCTGCCAGCCCAGAGAATT	GCACCCCCGATTTTTGC
NGF	ACACTCTGATCACTGCGTTTTTG	CCTTCTGGGACATTGCTATCTGT
P2X3	ACAAGATGGAGAATGGCAGCGAGT	TGATGTTGAACTTGCCAGCGTTCC
TRPV1	TTCCTGCAGAAGAGCAAGAAGC	CCCATTGTGCAGATTGAGCAT
IL1β	TAGGAGGCAGGTGGATTT	CCCTTTCCCTTTCCCTTTC
IL6	ACTGATGCTGGTGACAAC	CCGACTTGTGAAGTGGTATAG
TNFα	CCTATGTCTCAGCCTCTTCT	GGGAACTTCTCATCCCTTTG
MCP1	CACCTGCTGCTACTCATTC	CTACAGCTTCTTTGGGACAC
GFRα1	GTGTGCAGATGCTGTGGACTAG	TTCAGTGCTTCACACGCACTTG
TrkA	AGAGTGGCCTCCGCTTTGT	CGCATTGGAGGACAGATTCA
CaV2.2	TCCAGGAACAGGGAGATAAG	GTACTCAAAGGGTGGAGAGA
CAV3.2	GTTCCTGTCCATCATCACTC	GGGACTGTGACGTCTTATTG
Nav1.6	GACGGCTTCACCTTCTTAC	GCCTGGGATTACCGAAATAG
Nav1.7	GTGTTGGCTGGACTTCTT	GTTGACCACTACCCTCATTC
Nav1.8	GACACAACCTCGCTCTATTC	GGTTGTAAGGGTCGGATTG
Nav1.9	GCCCAACGAAGTGAAGAA	CCACGACCACACAATCAA

Supplementary Table 1: Primer information for gene tested using realtime PCR.

LTMR Mechanical Thresholds	
Control (n=2)	DhhCre;Nf1$^{f/f}$(n=8)
0.07 ± 0 g	0.07 ± 0 g
LTMR Mechanical Firing Rate	
Control (n=2)	DhhCre;Nf1$^{f/f}$(n=8)
12 ± 0 Hz	11 ± 2 Hz

Supplementary Table 2: Electrophysiological response properties of low threshold mechanoreceptors in control and DhhCre;Nf1$^{f/f}$ mice as determined using *ex vivo* recording. Although no differences were found between groups for any cell type analyzed here (1-way ANOVA on Ranks. p>0.05), low numbers recorded in control groups prevent firm conclusions.

Figure	Group Name	F and P Values	Stats test
Fig 1 A	Withdrawal threshold via R-S	F (1, 66) = 0.8081 P=0.3720	2-way RM ANOVA Tukey's post hoc
Fig 1 B	Withdrawal threshold via R-S	F (1, 99) = 0.7027 P=0.4039	2-way RM ANOVA Tukey's post hoc
Fig 1 C	Withdrawal threshold via R-S	F (1, 75) = 3.218 P=0.0769	2-way RM ANOVA Tukey's post hoc
Fig 1 D	Time spent in light chamber via MCA	F (2, 81) = 3.209 P=0.0456	1-way ANOVA with Tukey's post hoc
Fig 1 E	Time spent in light chamber via MCA	F (2, 75) = 3.087 P=0.0515	2-way RM ANOVA with HSD post hoc
Fig 1 F	Time spent in light chamber via MCA	F (2, 69) = 2.491 P=0.0131	1-way ANOVA with Tukey's post hoc
Fig 2 C	HTMR mechanical thresholds	F (2, 13) = 3.919 P=0.0466	1-way ANOVA with Tukey's post hoc
Fig 2 D	HTMR mechanical firing	F (2, 6) = 5.032 P=0.0521	1-way ANOVA with Tukey's post hoc
Fig 2 E	HTMR Heat thresholds	F (2, 2) = 1.516 P=0.03975	2-way ANOVA with Tukey's post hoc
Fig 2 F	CPM mechanical thresholds	F (2, 14) = 0.02276 P=0.478	1-way ANOVA with Tukey's post hoc
Fig 2 G	CPM mechanical firing	F (2, 10) = 12.41 P=0.0020	1-way ANOVA with Tukey's post hoc
Fig 3 H	CPM heat thresholds	F (2, 12) = 0.6204 P=0.5541	1-way ANOVA with Tukey's post hoc
Fig 3 A:v	Dissociated Schwann Cells	F (1, 75) = 156.4 P<0.0001	1-way ANOVA with Tukey's post hoc
Fig 3 B	Withdrawal threshold via R-S	F (1, 34) = 5.232 P=0.0285	2-way ANOVA with Tukey's post hoc
Fig 3 C	Time spent in light chamber via MCA	F (1, 10) = 3 P=0.0154	1-way ANOVA with Tukey's post hoc
Fig 3 D	CPM Mechanical Thresholds	F (26, 8) = 3.570 P=0.0333	1-way ANOVA with Tukey's post hoc
Fig 3 E	CPM Mechanical Firing	F (5, 139) = 2.985 P=0.0137	2-way RM ANOVA with HSD post hoc

Fig 4 B	Dissociated Schwann cells	F (1, 8) = 7365<br>P<0.0001	2-way ANOVA with HSD post hoc
Fig 4 C	Time spent in light chamber via MCA pre & post c21 injection	F (1, 15) = 0.8894<br>P=0.0484	2-way ANOVA with<br><br>Tukey’s post hoc
Fig 6 B	Fluorescence Intensity	F (6, 5) = 8.805<br>P=0.0305	1-way ANOVA with Tukey’s post hoc
Fig 6 C	Time spent in light chamber via MCA pre &post αGDNF injection	F (3, 100) = 2.832<br>P=0.0426	2-way ANOVA with Tukey's post hoc
SF 1 A	Withdrawal threshold via R-S	F (5, 44) = 4.786<br>P=0.0014	1-way ANOVA with Tukey's post hoc
SF 1 D	CM Firing Rates	F (1, 1) = 1.860<br>P=0.4028	1-way ANOVA with Tukey's post hoc
SF 1 E	CM Mechanical Thresholds	F (1, 1) = 1.000<br>P=0.5000	1-way ANOVA with Tukey's post hoc
SF 1 F	CPM Heat Thresholds	F (10, 7) = 0.4005<br>P= 0.9080	1-way ANOVA with Tukey's post hoc
SF 2 A	Time spent in light chamber via MCA	F (1, 104) = 3.247<br>P=0.0745	2-way ANOVA with Tukey’s post hoc
SF 2 E	No. of axons grouped into bundles	F (2, 30) = 4.144e-014<br>P<0.0001	2-way ANOVA with Tukey's post hoc
SF 3 D	AM Mechanical Thresholds	F (2, 6) = 0.007557<br>P=0.9925	1-way ANOVA with Tukey's post hoc
SF 3 E	AM Firing Rates	F (2, 6) = 1.962<br>P=0.2211	1-way ANOVA with Tukey's post hoc
SF 4	Time spent in light chamber via MCA pre & post c21 injection	F (1, 86) = 13.83<br>P=0.0004	2-way ANOVA with Tukey's post hoc<br>post hoc
SF 6 B	Fluorescence Intensity	F (1, 28) = 4.074<br>P=0.0532	2-way ANOVA with Tukey's post hoc
SF 6 C	Time spent in light chamber via MCA pre &post αGDNF injection	F (1, 100) = 0.04409<br>P=0.8341	2-way ANOVA with Tukey's post hoc

Supplementary Figure 3: Statistical information from data presented in figures of the manuscript.
